# Multifocal pancreatic PPoma in the setting of MEN1: Case report and review of literature

**DOI:** 10.1016/j.ijscr.2021.106008

**Published:** 2021-05-23

**Authors:** Arman Mosenia, Casey Ward, Alisa Yee, Amir Qorbani, Carlos Corvera

**Affiliations:** a School of Medicine, University of California San Francisco, San Francisco, CA, USA; b Department of Surgery, University of California San Francisco, San Francisco, CA, USA; c Department of Surgery, Division of Surgical Oncology, University of California San Francisco, San Francisco, CA, USA; d Department of Pathology, University of California San Francisco, San Francisco, CA, USA

**Keywords:** CgA, chromogranin A, ISGPS, International Study Group in Pancreatic Surgery, MEN1, multiple endocrine neoplasia, pNET, pancreatic neuroendocrine tumors, PPoma, pancreatic polypeptide-producing tumor, RAMP, radical antegrade modular pancreatosplenectomy, VIPoma, vasoactive intestinal polypeptide-secreting tumors, Case report, PPoma, MEN1, Multifocal, pNET, Ga68-DOTATATE PET/CT

## Abstract

**Introduction and importance:**

Functioning pancreatic neuroendocrine tumors (pNETs) that express pancreatic polypeptide—PPomas—do not yet have a pathognomonic clinical syndrome associated with them due to their overall rarity and diverse symptoms. Moreover, in patients with MEN1, the often multifocal nature of pNETs presents a unique clinical issue.

**Case presentation:**

We report a case of a 22-year-old man with a known *MEN1* gene mutation who was suffering from severe diarrhea (7–8 bowel movements per day) and was found to have only elevated PP levels on biochemical work-up. Ga68-DOTATATE PET/CT showed multifocal tumors in the body and tail of the pancreas that were not evident on contrast-enhanced CT. The patient underwent a successful laparoscopic radical antegrade modular pancreatosplenectomy (RAMP) and recovered well post-operatively with complete resolution of his diarrhea. Immunohistochemistry showed multiple pure PPomas.

**Clinical discussion:**

This case highlights the unique propensity for multifocal disease in patients with *MEN1* mutations and the utility of functional imaging by somatostatin analogs, i.e., Ga68-DOTATATE PET/CT, in order to perform oncologic laparoscopic pancreatic resections.

**Conclusion:**

PPomas in the setting of *MEN1* mutations are a unique clinical entity due to their diverse associated clinical syndromes and propensity for multifocal disease.

## Highlights

• PPomas have no pathognomonic clinical syndrome associated with them.
• Patients with MEN1 have a propensity for multifocal pNETs.
• DOTATE PET/CT is vital adjunct to pancreatic protocol CT for operative planning.
• Intra-operative ultrasound makes laparoscopic multi-focal pNET resection feasible.
• DOTATATE PET/CT surveillance is necessary due to high risk for recurrence in MEN1.

## 1 Introduction

Neuroendocrine tumors (NETs) are diverse neoplasms that most often originate in the gastrointestinal tract, lungs, and pancreas. Pancreatic NETs (pNETs), which account for 7% of all NETs, and stem from various cells within the islets of Langerhans [1], are relatively rare but their incidence has been increasing over the past decade, from 1–2 to 5–7 per 100,000 per year across North America [2,3]. pNETs range from low-grade indolent tumors to high-grade malignant tumors with high metastatic potential [4] and are further categorized into functioning or non-functioning based on their clinical manifestations. Functioning pNETs are named based on their predominant hormone-related syndrome, such as insulinomas, gastrinomas, vasoactive intestinal polypeptide-secreting tumors (VIPomas), glucagonomas, somatostatinomas, and other rare functional tumors including pancreatic polypeptide-producing tumor (PPoma).

Pure PPomas, immunostaining for PP only, are one of the rarest forms of pNETs with <15 cases reported to date [[5], [6], [7]]. PPomas arise from F cells, which are diffusely located within the uncinate and head of the pancreas and produce >90% of the body's pancreatic polypeptide (PP) [8]. PP is a regulatory substance that modulates somatostatin release and inhibits pancreas exocrine secretions. Consequently, PPomas have not been clearly associated with a clinical syndrome. Due to their quiescent progression, they are often found incidentally or at advanced stages due to mass effect, causing non-specific pain or hepatobiliary obstructive symptoms [9].

Although PPomas often occur sporadically, they have been associated with multiple endocrine neoplasia type 1 (MEN1). MEN1 syndrome is caused by a germline mutation in the *MEN1* gene, a tumor suppressor. MEN1 syndrome is traditionally defined by a triad of parathyroid, pancreatic and pituitary manifestations. MEN1 is inherited in an autosomal dominant fashion and may confer a varying combination of more than 20 endocrine and nonendocrine tumors, rarely known to include multifocal pNETs. The most common pNET in MEN1 is gastrinoma (~40%), with PPoma found in <2% of cases [10]. Consequently, the malignancy potential of PPomas in the setting of MEN1 is unclear. Herein, we report an unusual case of multifocal PPoma in the setting of MEN1. This case is reported in line with the Updating Consensus Surgical Case Report (SCARE 2020) guidelines [11].

## 2 Case presentation

**Fig. 1:**
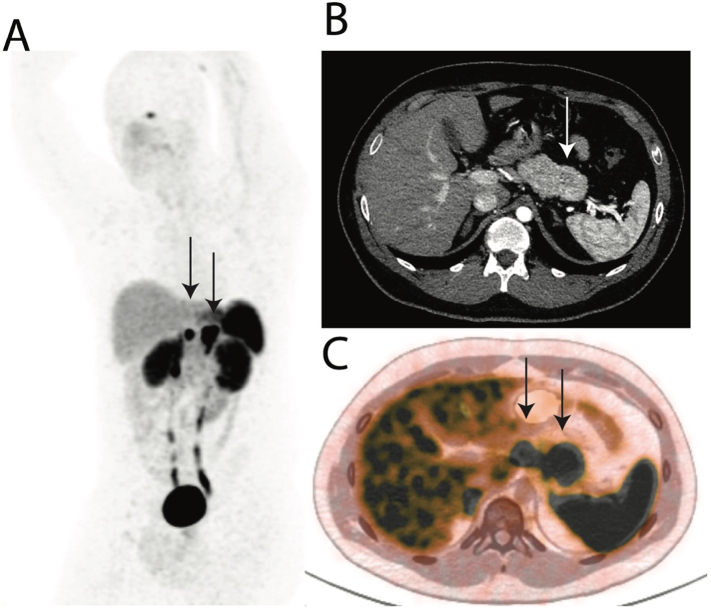
Pre-operative imaging showing two presumed pNETs within pancreatic body and tail. (A) Max Intensity Projection (MIP) of Ga68-DOTATATE PET/CT showing coronal projection of pancreatic body and tail lesions indicated by black arrows. (B) Late arterial-phase CT scan with only pancreatic tail lesion identified. (C) Fused 68Ga-DOTATE PET/CT identifying pancreatic body and tail lesions as seen on MIP.

A 22-year-old Caucasian man, with a known family history of MEN1 in his father and sister, was found to have a pathogenic mutation of MEN1 and first presented to our center for biochemical and radiological screening. He reported severe insomnia, weight gain despite loss of appetite, nausea without vomiting, abdominal pain, and longstanding diarrhea up to 7–8 times per day. He also reported heat intolerance, anxiety and depression. His past medical history was notable for recurrent nephrolithiasis since age 14, Attention Deficit Hyperactivity Disorder and remote history of heart palpitations. Elevated serum levels of calcium (10.6 mg/dL) and parathyroid hormone (64 pg/mL) indicated primary hyperparathyroidism. Parathyroid sestamibi scan was negative. Brain MRI showed no pituitary masses. Surveillance chest and abdominal CT imaging revealed a 3 mm nodule in the upper lobe of the right lung with benign features and a 2.2 cm pancreatic tail lesion with ill-defined enhancement concerning for pNET (Fig. 1B). Fasting pancreatic polypeptide was elevated at 574 pg/mL (normal range 56–480 pg/mL). Serum levels of the following were all within normal limits: insulin c-peptide 0.9 ng/mL (0.8–3.5 ng/mL), glucagon 56 pg/mL (8–57 pg/mL), gastrin <15 pg/mL, VIP <50 pg/mL, and interestingly, chromogranin A (CgA) 74 ng/mL (25–140 ng/mL). The patient was referred to tertiary academic medical center for operative evaluation by surgical oncology (author CC). A preoperative Ga68-DOTATATE PET/CT whole-body scan showed a pancreatic tail lesion measuring 2.1 × 1.2 cm and a pancreatic body lesion of 1.5 × 1.1 cm (Fig. 1A, C). Importantly, the latter was not detected on prior CT imaging. The increased sensitivity of Ga68-DOTATATE PET/CT allowed detection of this additional tumor [12]. Based on tumor locations, the patient was deemed a good surgical candidate for a laparoscopic radical antegrade modular pancreatosplenectomy (RAMP).

Exploratory laparoscopy revealed no evidence of metastatic disease. The pancreas was exposed, and intra-operative ultrasound used to confirm location of the two tumors within the body and tail previously identified radiographically. A laparoscopic RAMPs was performed to ensure negative margins. A reinforced stapler was used to divide the pancreas approximately 2 cm from the identified tumor at the level of the pancreatic neck. A regional lymph node dissection was done. Estimated blood loss was 50 cm^3^. Postoperative recovery was relatively uneventful outside of a low output biochemical leak, as defined by ISGPS (International Study Group in Pancreatic Surgery) [13], with drain amylase of 6366 U/L on post-operative day (POD) 3. He was discharged home with the drain in place on POD 3, which was removed on POD 12 after an interventional radiology tube study confirmed no fistula or collection. At 2 week follow-up, the patient had recovered well and reported complete resolution of his diarrhea that has persisted out to latest 6 month follow-up.

**Fig. 2:**
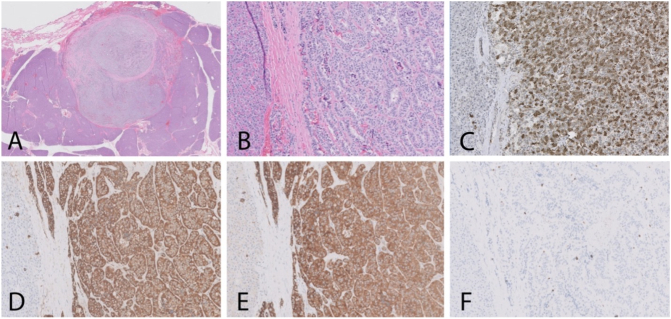
(A) H&E sections (40× magnification) show a well-circumscribed mass with a fibrous pseudocapsule in pancreas. (B) H&E sections (200× magnification) show small, uniform, polygonal and cuboidal cells with round to oval nuclei, stippled chromatin (“salt and pepper”), and lightly eosinophilic to finely granular cytoplasm, arranged in nests and trabeculae. (C) Pancreatic polypeptide immunostain (200× magnification) shows positive immunoreactivity in the tumor compared to the adjacent normal pancreatic tissue. (D) Chromogranin immunostain (200× magnification) shows positive immunoreactivity in tumor cells. (E) Synaptophysin immunostain (200× magnification) shows positive immunoreactivity in tumor cells. (F) Ki-67 immunostain (200× magnification) shows scattered positive cells; 3–5% proliferative index.

The final pathology report revealed five well-differentiated grade 2 NETs ranging from 0.1 to 1.7 cm with 3 mitotic figures/10 high-power fields and Ki-67 index of 3–5% based on semi-quantitative immunohistochemical stain (Fig. 2F). Margins were negative. Twelve lymph nodes were examined; none showed evidence of tumor. AJCC Pathologic Stage was determined to be pT1(m)N0. Tumors stained positive for Synaptophysin (Fig. 2E), Chromogranin (Fig. 2D), and PanCK. Additional staining confirmed strong positive staining for PP (Fig. 2C).

## 3 Discussion

Our patient's case highlights a unique presentation of multi-focal pancreatic PPoma in the setting of a known *MEN1* genetic mutation with complete resolution of longstanding diarrhea after resection.

A characteristic feature of pNETs in MEN1 is the presence of multi-focal intraparenchymal pancreatic tumors, in contrast to the solitary pNETs found in sporadic cases [14,15]. The finding of microadenomatosis, defined by the presence of multiple pNETs measuring ≤0.5 cm, or microadenomas mixed with macroadenomas (>1 cm), is present in almost all MEN1 cases [14,15].

Our patient's clinical manifestation of severe diarrhea that resolved with resection is unique among published cases of pure multifocal PPoma. Historically, PP-secreting tumors were thought to cause severe diarrhea but conflicting PPoma case reports subsequently showed only a third of published cases documenting diarrhea [16]. Thus, the heterogenous symptoms of documented PPomas have not yet resulted in an identifiable clinical syndrome and are often managed clinically as non-functional pNETs.

To date, approximately 30 patients with PPomas have been reported, 13 with histopathologic confirmation of pure PPomas (Supplementary Table 1). Even rarer is the presence of pure, multifocal PPoma in the setting of MEN1, as in our patient. Because of this rarity, the malignant and metastatic potential of PPoma in the setting of MEN1 is unknown. PPomas have been thought to confer higher local recurrence and distal metastases rates, but that may be due to the non-functioning nature of the tumor and difficulty in diagnosing it, rather than to tumor biology [9]. For instance, previous studies sought to ascertain if a 2 cm cutoff for resection candidacy used in sporadic pNETs should apply to pNETs in MEN1 patients. A study with >10 years of follow-up data for 46 patients with MEN1 and non-functioning pNETs (NF-pNETs) ≤ 2 cm who did not have surgery at the time of diagnosis reported that 28 (61%) patients had stable disease and 16 had progression, seven of whom required surgery [17]. One patient died of metastatic disease, but none of the living patients had evidence of metastatic disease at last follow-up. The authors concluded that conservative management without surgery in patients with MEN1 and NF-pNETs measuring ≤2 cm results in low disease-specific mortality [17]. The number of PPomas that may have been included as NF-pNETs in that study is unknown.

Because surveillance and early diagnosis may decrease the morbidity and mortality, biochemical markers such as serum CgA and PP are important to include in all MEN1 and potential pNET evaluations. Increased levels of serum CgA, PP and pancreastatin combined have a sensitivity of up to 95% [8]. Additionally, as illustrated in our patient, functional imaging by somatostatin analogs, i.e., Ga68-DOTATATE PET/CT, refines the diagnostic sensitivity of preoperative identification of multifocal pNETs.

Although our patient had no radiographic evidence of additional intra-pancreatic or distant metastatic disease, he remains at significant risk for additional or recurrent disease due to his known multifocal disease and *MEN1* mutation and will require close follow-up. At our center, we recommend bi-annual Ga68-DOTATATE PET/CT surveillance scans for the first 5 years and then yearly. Careful surveillance for metastatic disease within the liver is crucial because debulking and metastasectomy have improved survival in patients with pNETs [18,19]. The role of systemic therapy, specifically somatostatin analogs, in patients with pNETs and MEN1 mutations remains controversial, with ongoing studies underway to assess their potential suppressive utility long-term [20].

## 4 Conclusion

Herein, we report a case of a young patient with multifocal PPoma causing severe diarrhea in the setting of MEN1. Screening with biologic markers of pNETs and Ga68-DOTATATE PET/CT imaging, provided timely diagnosis, enabling R0 resection of the tumors by laparoscopic IOUS and distal RAMPs. We recommend complete immunohistochemistry with complete histological hormonal markers post-operatively to confirm diagnosis. Post-operative surveillance pancreatic imaging of MEN1 patients is crucial, especially since nonfunctioning pNETs like PPoma are known to grow quiescently. Our patient had significant improvement in his quality of life with laparoscopic distal RAMPs and will be followed closely by a multi-disciplinary team.

The following are the supplementary data related to this article.

Supplementary Table 1 Literature review of PPomas, confirmed by immunohistochemistry, described in the literature to date. *Some cases within this report were excluded as no confirmatory immunohistochemistry was provided. Ga = gastrin, Glu = glucagon, In = insulin, PP = polypeptide, SS = somatostatin, VIP = vasoactive intestinal peptide, DM = diabetes mellitus, RFA = radiofrequency ablation. Supplementary Table 1

## Consent

Written informed consent was obtained from the patient for publication of this case report and accompanying images. A copy of the written consent is available for review by the Editor-in-Chief of this journal on request.

## Provenance and peer review

Not commissioned, externally peer-reviewed.

## Ethical approval

Not applicable, and patient data is de-identified.

## Funding

This research did not receive any specific grant from funding agencies in the public, commercial, or not-for-profit sectors.

## Guarantor

CC.

## Research registration number

Not applicable.

## CRediT authorship contribution statement

AM, CW, and CC jointly designed the study. CW and CC performed the surgery. CW, CC, AY and AM contributed to patient management. AM and AY performed data and evidence collection, respectively. AQ reviewed the pathology specimens. AM and CW created the initial draft of the manuscript. All authors discussed the results and contributed to manuscript revision.

## Declaration of competing interest

None.
