# Supplementary material for: Multifocal pancreatic PPoma in the setting of MEN1: Case report and review of literature
Source: Int J Surg Case Rep. 2021 May 23;83:106008. doi: 10.1016/j.ijscr.2021.106008 (PMC8193151; doi:10.1016/j.ijscr.2021.106008)
Supplement: Supplementary Table 1 — Literature review of PPomas, confirmed by immunohistochemistry, described in the literature to date. *Some cases within this report were excluded as no confirmatory immunohistochemistry was provided. Ga = gastrin, Glu = glucagon, In = insulin, PP = polypeptide, SS = somatostatin, VIP = vasoactive intestinal peptide, DM = diabetes mellitus, RFA = radiofrequency ablation. [file mmc1.docx]

| **Paper** | **Year** | **Age/**  **Sex** | **MEN1** | **Pure Ppoma** | **Histology ("+" Positive, "-" Negative)** | **Multifocal (Location)** | **Max**  **Diameter**  **(cm)** | **Described Symptoms** | **Metastasis (Location)** | **Surgical Treatment** | **Oncologic Outcome (at publication)** |
| --- | --- | --- | --- | --- | --- | --- | --- | --- | --- | --- | --- |
| Bordi^1^ | 1978 | 45M | n/a | N | "+" PP | Y (body and tail) | 0.8 | Recurrent duodenal ulcer, weight loss | N | Billroth II, enucleation | Alive @ 1 yr |
|  |  |  |  |  | "-" VIP, Ga, In, Glu, SS |  |  |  |  |  |  |
| Friesen^2^ | 1980 | 33 M | Y | N | "+" PP | N (tail) | 12 | Asymptomatic | N | Distal pancreatectomy | Alive @ 2yrs |
|  |  |  |  |  | "weak +" In, Glu, SS |  |  |  |  |  |  |
|  |  | 29 F | Y | N | "+" PP, Ga | n/a | n/a | Asymptomatic | Y | Distal pancreatectomy, partial duodenectomy | n/a |
|  |  | n/a | N | N | "+" PP, Ga | n/a | n/a | Duodenal ulcer | n/a | Distal pancreatectomy | Alive |
|  |  | n/a | N | N | "+" PP, Ga | n/a | n/a | Gastric ulcer, diarrhea | n/a | Vagotomy with antrectomy, biopsy | n/a |
| Hayes^3^ | 1980 | 55M | n/a | Y | "+" PP | N (body) | 5 | WHDA, weight loss | N | Supportive | Dead |
|  |  |  |  |  | "-" VIP, In, Glu, SS |  |  |  |  |  |  |
| Nobin^4^ | 1984 | 37M | N | N | "+" PP, Gastrin | N (tail) | n/a | Abdominal pain, weight loss, diarrhea | Y (liver) | Supportive | Dead |
|  |  | 57F | N | Y | "+" PP | N (Head) | n/a | Abdominal pain | Y (Liver, LN) | Laparotomy, biopsy | Alive @ 2yrs |
|  |  |  |  |  | "-" VIP, Ga, In, Glu, SS |  |  |  |  |  |  |
| Strodel^5^ | 1984 | 53M | N | Y | "+" PP | N (Head) | 5 | Epigastric pain, gastritis | n/a | Whipple | n/a |
|  |  |  |  |  | "-" VIP, Ga, In, Glu, SS |  |  |  |  |  |  |
|  |  | 74F | N | N | "+" PP, VIP | N (body) | 7 | Weight loss, diarrhea | N | Subtotal pancreatectomy | Alive |
|  |  |  |  |  | "-" Ga, In, Glu, SS |  |  |  |  |  |  |
|  |  | 56M | N | Y | "+" PP | N (Head) | 1.5 | Abdominal pain | n/a | Whipple | n/a |
|  |  |  |  |  | "-" VIP, Ga, In, Glu, SS |  |  |  |  |  |  |
|  |  | 20F | N | Y | "+" PP | N (Head) | 15 | Weight loss, jaundice | N | Whipple | Alive @ 3yrs |
|  |  |  |  |  | "-" VIP, Ga, In, Glu, SS |  |  |  |  |  |  |
|  |  | 63F | Y | Y | "+" PP | Y (diffuse) |  | Weight loss | N | Subtotal pancreatectomy, enucleation | Alive |
|  |  |  |  |  | "-" VIP, Ga, In, Glu, SS |  |  |  |  |  |  |
|  |  | 52M | Y | N | "+" PP, In, Glu, SS "-" Ga, VIP | Y (diffuse) | n/a | Abdominal pain, peptic ulcer disease | n/a | Whipple | n/a |
|  |  | 67M | N | Y | "+" PP | Y (diffuse) | n/a | Weight loss | n/a | Supportive | n/a |
|  |  |  |  |  | "-" VIP, Ga, In, Glu, SS |  |  |  |  |  |  |
|  |  | 48M | N | N | Not described | N (tail) | 4 | Weight loss, GI bleeding | Y (Liver) | Exploration | Dead |
| Tomita*^6^ | 1986 | 26M | Y | N | "+" PP | N (tail) | 15 | Asymptomatic | Y (Liver, bone) | Distal pancreatectomy | Dead @ 3yrs |
|  |  |  |  |  | "weak +" In |  |  |  |  |  |  |
| Choksi^7^ | 1988 | 59F | N | Y | "+" PP | N | 10 | GI bleed, skin rash | Y (Liver) | Debulking, chemo | Alive @ 1yr |
|  |  |  |  |  | "-" VIP, In, Glu, SS |  |  |  |  |  |  |
| Guiro^8^ | 1994 | 67F | n/a | Y | "+" PP | N (tail) | 10 | Asymptomatic | N | Distal pancreatectomy | Dead |
|  |  |  |  |  | "-" VIP, Ga, In, Glu, SS |  |  |  |  |  |  |
|  |  | 66F | n/a | Y | "+" PP | N (tail) | 10 | Abdominal pressure | Y (LN) | Total pancreatectomy | Alive @ 6yrs |
|  |  |  |  |  | "-" VIP, Ga, In, Glu, SS |  |  |  |  |  |  |
| Mehring^9^ | 1997 | 58F | Y | N | "+" PP and SS | Y (diffuse) | 2.5 | Peptic ulcers | N | Subtotal pancreatectomy | Alive @ 6yrs |
|  |  |  |  |  | "-" In, Glu, Ga, VIP |  |  |  |  |  |  |
| Bellows^10^ | 1998 | 75 F | N | Y | "+" PP | N (tail) | 3.7 | Abdominal pain | N | Distal pancreatectomy and splenectomy | Alive @ 0.7yrs |
|  |  |  |  |  | "-" In, Glu, SS, Ga. |  |  |  |  |  |  |
| Mortenson^11^ | 2002 | 46 F | Y | Y | "+" PP, VIP, Glu, Ga, SS | N (tail) | 0.6 | Watery diarrhea | N | Distal pancreatectomy and splenectomy | Alive |
|  |  |  |  |  | "-" In |  |  |  |  |  |  |
| Colovic^12^ | 2003 | 19F | n/a | N | "+" PP | Y (diffuse) | 6 | Recurrent pancreatic fistula | Y (Liver) | Whipple | Dead @ 3yrs |
|  |  |  |  |  | "weak +" Glu, SS |  |  |  |  |  |  |
|  |  |  |  |  | "-" In, Ga |  |  |  |  |  |  |
|  |  | 55 F | n/a | N | "+" PP, SS | N (Head) | 6 | Epigastric pain, weight loss | N | Whipple | Alive @ 6m |
|  |  |  |  |  | "weak +" VIP |  |  |  |  |  |  |
| Raffel^13^ | 2004 | 65F | N | Y | "+" PP | N (Head) | 9 | Gastric ulcer | N | Whipple | Alive @8 yrs |
|  |  |  |  |  | "-" Ga, Glu, In |  |  |  |  |  |  |
| Rossi^14^ | 2006 | 25 F | N | N | "+" PP, VIP | N (tail) | 3.7 | Severe asthenia and myalgias | N | Subtotal pancreatectomy | Alive @4 yrs |
|  |  |  |  |  | "-" In, Glu, SS |  |  |  |  |  |  |
| Kuo^15^ | 2008 | 79F | N | N | "+" PP, Ga "weak +" SS | N (Head) | 1 | Abdominal pain | N | Whipple | Alive @3 yrs |
|  |  |  |  |  | "-" In |  |  |  |  |  |  |
|  |  | 59M | N | N | "+" PP, SS | N (Uncinate) | 2.5 | Abdominal pain | N | Enucleation | Alive @3 yrs |
|  |  |  |  |  | "-" Ga,In |  |  |  |  |  |  |
|  |  | 31M | N | N | "+" PP, "weak +" In | N (Head) | 2.2 | Abdominal pain | N | Enucleation | Alive @3 yrs |
|  |  |  |  |  | "-" SS, Ga. |  |  |  |  |  |  |
|  |  | 48F | N | Y | "+" PP | N (Neck) | 2.4 | Abdominal pain, Diarrhea | N | Distal pancreatectomy | Alive @3 yrs |
|  |  |  |  |  | "-" In, Ga, SS |  |  |  |  |  |  |
| Amrilleva^16^ | 2008 | 46F | N | Y | "+" PP | N (Head) | 3 | Diarrhea | N | Enucleation, pancreaticojejunostomy | Alive @3 yrs |
|  |  |  |  |  | "-" VIP, Ga, In, Glu, SS |  |  |  |  |  |  |
| Maxwell*^17^ | 2014 | 52M | N | N | "+" PP | N (Head) | 7.4 | Weight loss, new DM | N | Whipple | Alive @ 1 yr |
|  |  | 68M | N | N | "+" PP | N (tail) | 2.6 | Chest pain | N | Distal pancreatectomy, splenectomy, cholecystectomy | Alive @ 20 m |
|  |  | 42F | Y | N | "+" PP | Y (head/body) | 0.8 | Epigastric pain, nausea, vomiting | Y (liver) | Enucleation, liver wedge resection, RFA | Alive @ 3.5 yrs |

Supplementary Table 1: Literature review of PPomas, confirmed by immunohistochemistry, described in the literature to date. *Some cases within this report were excluded as no confirmatory immunohistochemistry was provided. Ga=Gastrin, Glu=Glucagon, In=Insulin, PP=polypeptide, SS=somatostatin, VIP=Vasoactive Intestinal Peptide, DM= Diabetes Mellitus, RFA= Radiofrequency Ablation

1 Bordi, C. *et al.* Human islet cell tumor storing pancreatic polypeptide: a light and electron microscopic study. *J Clin Endocrinol Metab* **46**, 215-219, doi:10.1210/jcem-46-2-215 (1978).

2 Friesen, S. R., Kimmel, J. R. & Tomita, T. Pancreatic polypeptide as screening marker for pancreatic polypeptide apudomas in multiple endocrinopathies. *Am J Surg* **139**, 61-72, doi:10.1016/0002-9610(80)90231-7 (1980).

3 Hayes, M. M. Report of a pancreatic polypeptide-producing islet-cell tumour of the pancreas causing the watery diarrhoea, hypokalaemia, achlorhydria syndrome in a 55 year old Zimbabwean African male. *Cent Afr J Med* **26**, 195-197 (1980).

4 Nobin, A. *et al.* Pancreatic polypeptide-producing tumors. Report on two cases. *Cancer* **53**, 2688-2691, doi:10.1002/1097-0142(19840615)53:12<2688::aid-cncr2820531221>3.0.co;2-v (1984).

5 Strodel, W. E. *et al.* Pancreatic polypeptide-producing tumors. Silent lesions of the pancreas? *Arch Surg* **119**, 508-514, doi:10.1001/archsurg.1984.01390170008003 (1984).

6 Tomita, T., Friesen, S. R. & Kimmel, J. R. Pancreatic polypeptide-secreting islet cell tumor. A follow-up report. *Cancer* **57**, 129-133, doi:10.1002/1097-0142(19860101)57:1<129::aid-cncr2820570126>3.0.co;2-q (1986).

7 Choksi, U. A., Sellin, R. V., Hickey, R. C. & Samaan, N. A. An unusual skin rash associated with a pancreatic polypeptide-producing tumor of the pancreas. *Ann Intern Med* **108**, 64-65, doi:10.7326/0003-4819-108-1-64 (1988).

8 Fuertes Guiró, F., Mortara, G., Schiaffino, E. & d'Urbano, C. [Pancreatic endocrine F-cell tumor]. *Rev Esp Enferm Dig* **86**, 694-698 (1994).

9 Mehring, U. M., Jäger, H. J., Klöppel, G. & Hasse, F. M. [Pancreatic polypeptide secreting endocrine pancreas tumor associated with multiple stomach and duodenal ulcers]. *Langenbecks Arch Chir* **382**, 134-137 (1997).

10 Bellows, C., Haque, S. & Jaffe, B. Pancreatic polypeptide islet cell tumor: case report and review of the literature. *J Gastrointest Surg* **2**, 526-532, doi:10.1016/s1091-255x(98)80052-8 (1998).

11 Mortenson, M. & Bold, R. J. Symptomatic pancreatic polypeptide-secreting tumor of the distal pancreas (PPoma). *Int J Gastrointest Cancer* **32**, 153-156, doi:10.1385/IJGC:32:2-3:153 (2002).

12 Colović, R. *et al.* [Two cases of pancreatic head polypeptide tumors, one with a central cavity which fistulized into the duodenum]. *Srp Arh Celok Lek* **131**, 259-265, doi:10.2298/sarh0306259c (2003).

13 Raffel, A., Krausch, M., Schulte, K. M. & Röher, H. D. Symptomatic pure pancreatic polypeptide-containing tumor of the pancreas. *Pancreas* **29**, 83, doi:10.1097/00006676-200407000-00060 (2004).

14 Rossi, V. *et al.* Hypokalemic rhabdomyolysis without watery diarrhea: an unexpected presentation of a pancreatic neuro-endocrine tumor. *Am J Gastroenterol* **101**, 669-672, doi:10.1111/j.1572-0241.2006.00392.x (2006).

15 Kuo, S. C., Gananadha, S., Scarlett, C. J., Gill, A. & Smith, R. C. Sporadic pancreatic polypeptide secreting tumors (PPomas) of the pancreas. *World J Surg* **32**, 1815-1822, doi:10.1007/s00268-008-9499-7 (2008).

16 Amrilleva, V., Slater, E. P., Waldmann, J., Bonorden, D. & Fendrich, V. A Pancreatic Polypeptide-Producing Pancreatic Tumor Causing WDHA Syndrome. *Case Rep Gastroenterol* **2**, 238-243, doi:10.1159/000142739 (2008).

17 Maxwell, J. E., O'Dorisio, T. M., Bellizzi, A. M. & Howe, J. R. Elevated pancreatic polypeptide levels in pancreatic neuroendocrine tumors and diabetes mellitus: causation or association? *Pancreas* **43**, 651-656, doi:10.1097/MPA.0000000000000082 (2014).
